# Supplementary material for: Believing that difficulty signals importance improves school outcomes by bolstering academic possible identities, a recursive analysis
Source: PLoS One. 2024 Oct 21;19(10):e0308376. doi: 10.1371/journal.pone.0308376 (PMC11493252; doi:10.1371/journal.pone.0308376)
Supplement: S1 File — (DOCX) [file pone.0308376.s001.docx]

**SUPPLEMENTAL MATERIALS**

**Sample Description**

Table S1 shares a detailed, descriptive snapshot of our sample. On average, students’ favorite subject was math. They spent over an hour daily on homework, tutoring, reading, and sports and less than an hour on physical education, the internet, television, video games, and music or dance. Before the high-stakes test, students reported working moderately hard in school, believed they had a modest chance of getting into their ideal school, had high hopes that this would occur, and were modestly worried they would not.

On the day students learned their high-stakes test results, we asked them to what extent their test scores were due to ability, effort, luck, and fate. Students' attributions about the reason for their scores were distinct (see Supplemental Materials Table S3 for correlations). Across linear regression models controlling for high-stakes test scores (see Supplemental Materials Table S4 for detailed results), students' attributions about the reason for their scores were associated with their T1 metacognitive inferences from difficulty and culture-based beliefs. Thus, students were more likely to believe their test scores were due to their ability if at T1 they endorsed difficulty-as-importance more. Students were more likely to believe their test scores were due to their effort if at T1 they endorsed difficulty-as-importance and optimism for the future more and difficulty-as-impossibility less. They were more likely to believe their test scores were due to luck if at T1 they endorsed difficulty-as-impossibility and accepting fate more and optimism for the future less. They were more likely to believe their test scores were due to fate if at T1 they endorsed difficulty-as-importance and optimism for the future less and difficulty-as-impossibility and accepting fate less. We take these distinct patterns of associations as suggesting that difficulty-as-importance, difficulty-as-impossibility, optimism for the future, and accepting fate each may play a unique, motivational role in how student’s make sense of their academic outcomes.

**Table S1***Snapshot of the Sample Descriptive Results: Means and Standard Deviations (SD).*

| Variable | Mean (%, Hours, or Score) | *SD* |
| --- | --- | --- |
| % Chose Math as Favorite Subject | 35.69% | 47.95 |
| Daily Activities (in hours) | | |
| Doing Homework | 1.84 | 0.74 |
| Receiving Tutoring | 1.31 | 1.20 |
| Reading | 1.17 | 0.83 |
| Playing Sports | 1.08 | 0.74 |
| In PE | 0.94 | 0.54 |
| Using the Internet | 0.77 | 0.78 |
| Watching Television | 0.76 | 0.76 |
| Playing Video Games | 0.63 | 0.78 |
| Playing Music or Dancing | 0.58 | 0.71 |
| Belief and Attribution Items (responses from 0 to 9 except as noted) | | |
| 6^th^-Grade Effort (1 to 10) | 6.51 | 1.71 |
| Likely to Get into Ideal School | 5.16 | 2.29 |
| Hope to Get into Ideal School | 7.76 | 1.98 |
| Worry if Get into Ideal School | 5.75 | 2.78 |
| Ability Impacts Exam Score | 5.60 | 2.34 |
| Effort Impacts Exam Score | 6.59 | 2.15 |
| Luck Impacts Exam Score | 3.76 | 2.52 |
| Fate Impacts Exam Score | 3.16 | 2.69 |

**Test-Score Attributions of Ability, Effort, Luck, and Fate**

In Table S2, we present correlations between students’ attributions about the reason for their high-stakes test scores.

**Table S2**

*Bivariate Correlations Between Test-Score Attributions*

|  | Ability | Effort | Luck | Fate |
| --- | --- | --- | --- | --- |
| Ability | -- | .53 | .10 | .00 |
| Effort | <.001 | -- | .08 | -.09 |
| Luck | .055 | .118 | -- | .41 |
| Fate | .971 | .089 | <.001 | -- |

*Note.* Pearson correlation coefficients are presented above the diagonal and *p*-values are presented below the diagonal.

We also ran a series of linear regression models to explore whether our measures of metacognitive inferences from difficulty and culture-based beliefs at T1 predicted test-score attributions at T2. We controlled for high-stakes test scores in each regression model. We present the detailed results from these 16 regression models in Table S3.

**Table S3**

*Predicting T2 Test-Score Attributions from T1 Metacognitive Inferences from Difficulty and Culture-Based Beliefs*

| Outcome Variable | Effect | *B* | *SE B* | *t* | *p* |
| --- | --- | --- | --- | --- | --- |
| T2 Ability Attribution | T1 Difficulty-as-Importance | 0.26 | 0.13 | 1.98 | .048 |
|  | T1+ High-Stakes Test Scores | 1.07 | 0.22 | 4.76 | <.001 |
| T2 Ability Attribution | T1 Difficulty-as-Impossibility | -0.09 | 0.13 | -0.73 | .465 |
|  | T1+ High-Stakes Test Scores | 1.14 | 0.23 | 4.90 | <.001 |
| T2 Ability Attribution | T1 Accepting Fate | 0.05 | 0.12 | 0.40 | .692 |
|  | T1+ High-Stakes Test Scores | 1.23 | 0.23 | 5.43 | <.001 |
| T2 Ability Attribution | T1 Optimism for the Future | 0.17 | 0.10 | 1.65 | .099 |
|  | T1+ High-Stakes Test Scores | 1.11 | 0.22 | 5.05 | <.001 |
| T2 Effort Attribution | T1 Difficulty-as-Importance | 0.32 | 0.12 | 2.62 | .009 |
|  | T1+ High-Stakes Test Scores | 1.27 | 0.20 | 6.33 | <.001 |
| T2 Effort Attribution | T1 Difficulty-as-Impossibility | -0.25 | 0.11 | -2.20 | .028 |
|  | T1+ High-Stakes Test Scores | 1.26 | 0.21 | 6.06 | <.001 |
| T2 Effort Attribution | T1 Accepting Fate | -0.16 | 0.11 | -1.47 | .141 |
|  | T1+ High-Stakes Test Scores | 1.34 | 0.20 | 6.56 | <.001 |
| T2 Effort Attribution | T1 Optimism for the Future | 0.21 | 0.09 | 2.31 | .021 |
|  | T1+ High-Stakes Test Scores | 1.32 | 0.20 | 6.65 | <.001 |
| T2 Luck Attribution | T1 Difficulty-as-Importance | -0.25 | 0.15 | -1.73 | .084 |
|  | T1+ High-Stakes Test Scores | -0.14 | 0.25 | -0.58 | .564 |
| T2 Luck Attribution | T1 Difficulty-as-Impossibility | 0.35 | 0.14 | 2.55 | .011 |
|  | T1+ High-Stakes Test Scores | -0.03 | 0.25 | -0.10 | .921 |
| T2 Luck Attribution | T1 Accepting Fate | 0.43 | 0.13 | 3.23 | .001 |
|  | T1+ High-Stakes Test Scores | -0.01 | 0.25 | -0.06 | .956 |
| T2 Luck Attribution | T1 Optimism for the Future | -0.32 | 0.11 | -2.88 | .004 |
|  | T1+ High-Stakes Test Scores | -0.10 | 0.24 | -0.43 | .670 |
| T2 Fate Attribution | T1 Difficulty-as-Importance | -0.38 | 0.15 | -2.44 | .015 |
|  | T1+ High-Stakes Test Scores | -0.91 | 0.26 | -3.52 | <.001 |
| T2 Fate Attribution | T1 Difficulty-as-Impossibility | 0.77 | 0.14 | 5.36 | <.001 |
|  | T1+ High-Stakes Test Scores | -0.57 | 0.26 | -2.16 | .031 |
| T2 Fate Attribution | T1 Accepting Fate | 1.29 | 0.13 | 9.73 | <.001 |
|  | T1+ High-Stakes Test Scores | -0.33 | 0.25 | -1.32 | .186 |
| T2 Fate Attribution | T1 Optimism for the Future | -0.95 | 0.11 | -8.45 | <.001 |
|  | T1+ High-Stakes Test Scores | -0.59 | 0.24 | -2.42 | .016 |

*Note. B=*unstandardized coefficient. *SE B=*standard error of unstandardized coefficient.

**Scale Items**

Our scales were administered in Chinese. The Chinese and English translations for each scale are shared below. Each scale was answered using a *1 = strongly disagree to a 5 = strongly disagree* scale, except for the certainty scales which were answered using *a 0 = not possible at all to 9 = very possible* scale. Items marked with an asterisk (*) were dropped to ensure proper model fit. Items marked with (R) were reverse-scored items. Items marked with (F) were filler items.

***Certainty of Attaining Academic Possible Identities***

Chinese

1. 将来，我在学校表现优良.
2. 将来，我考试成绩好.
3. 将来，我理解课堂上的内容.

English

1. In the future, I will perform well in school.
2. In the future, I will have good grades.
3. In the future, I will understand the materials in class.

***Certainty of Using Effective Strategies to Attain Academic Possible Identities***

Chinese

1. 将来，我会合理安排时间.
2. 将来，我会成功解决遇到的困难.
3. 将来，我会处理好让我分心的事情.
4. 将来，我会朝着目标不懈努力.

English

1. In the future, I will manage my time.
2. In the future, I will successfully resolve the difficulties I encounter.
3. In the future, I will take care of the things that distract me.
4. In the future, I will work tirelessly toward my goal.

***Accepting Fate***

Chinese

1. 人的一切在出生时就已经由上天安排好了，是难以改变的.
2. 富贵贫贱，成败得失，都是命中注定的.
3. 没有实现自己的理想，主要是因为我没有那个命罢了.
4. 我相信命运掌握在自己手里. (R) *
5. 人生中有许多事只能服从命运的安排.
6. 我很少努力去改变一些事情，因为它们是命中注定改变不了的.
7. 即使是那些非常优秀的人，他们也只能服从命运的安排.
8. 只要努力就能改变个人的命运. (R) *
9. 有些事不是我自己能左右的，只能听从命运的安排.
10. 做事能否成功，取决于上天的安排.

English

1. God arranged everything from birth; it is difficult to change.
2. Rich or poor, success or failure is predestined.
3. I have not realized my ideals, mainly because it is not my fate to do so.
4. I believe that fate is in my own hands. (R) *
5. Many things in life cannot help but obey the arrangements of fate.
6. I rarely try to change things, they are as they are because they are not destined to change.
7. Even if one is quite excellent, fate is a determining factor in life’s outcomes.
8. As long as you work hard, you can change your destiny. (R) *
9. There are things I have no influence over, I can only obey my fated destiny.
10. God’s plans determine whether things succeed or not.

***Optimism for the Future***

Chinese

1. 放松对我来说很容易. (F)
2. 对我来说保持忙碌状态很重要. (F)
3. 我做事总是感到不顺利. (R)
4. 当事情出现问题时，我通常预料事情会有所好转.
5. 我觉得好事很少会发生在我身上. (R)
6. 我认为我会是个幸运的人.
7. 即便有好事发生，机会也不会落在我头上. (R)
8. 我每天都会过得很开心. (F)
9. 不管我怎么努力，我想事情都不会顺利发展. (R)
10. 总的来说，我认为将来发生在我身上的好事会比坏事多.

English

1. It’s easy for me to relax. (F)
2. It’s important for me to keep busy. (F)
3. I always feel uneasy when doing things. (R)
4. When things go wrong, I usually expect things to get better.
5. I feel like good things rarely happen to me. (R)
6. I think I will be a lucky person.
7. Even if something good happens, the opportunity will not fall on me. (R)
8. I will have a great time every day. (F)
9. No matter how hard I try, I don't think things will go smoothly. (R)
10. Overall, I expect more good things than bad things will happen to me in the future.

***Difficulty-as-importance***

Chinese

1. 学习中出现的种种困难，是在提醒我自己要努力钻研.
2. 考试成绩不理想，是在提醒我更加投入到学习中去.
3. 当我发现自己在学习上遇到困难时，这可能只是意味着学习对我而言是重要的.
4. 学习上的感受可以告诉我学习（学业）对我的重要性。如果我感到学习很难，但是仍能坚持下去，表明学习对我来说是件重要的事.
5. 作为一个学生，我明白：困难的目标就是重要的目标. 如果在学习过程中出现了困难，就意味着我要更努力.
6. 要取得良好的学生成绩，毫无疑问需要真正的刻苦努力. 那些需要我真正努力去做的事，意味着这些事对我很重要.

English

1. Difficulties in studying remind me to study hard.
2. Unsatisfactory test results remind me to devote myself to studying.
3. When I find myself encountering difficulties in learning, it may just mean that learning is important to me.
4. My feelings about learning can tell me how important learning (academics) is to me. If I find it difficult to learn, but I can persist, that shows me that learning is an important thing for me.
5. As a student, I understand that difficult goals are important goals. If there are difficulties in the learning process, that means I have to work harder.
6. To achieve good student results, there is no doubt that real hard work is required. Things that require me to really work hard are things that are important to me.

***Difficulty-as-impossibility***

Chinese

1. 人生中有许多事只能服从命运的安排.
2. 考试成绩不理想，是在提醒我自己不是读书的料.
3. 当我在学习中遇到困难时，这可能意味着我不擅长学习，转而做其他的事会更好一些.
4. 有时，感到学习太难了——学好甚至是件不可能的事. 这可能是件好事，因为这让我明白自己应该去做其他的事.
5. 学生不应该把时间浪费在对自己没有意义的事情上，如果感觉某件事太难了，就应该尝试做那些自己能做成功的事. *
6. 作为一个学生，如果我觉得学习太难的话，这可能意味着我不太适合学习.

English

1. Difficulties in studying remind me that I am not good at reading.
2. Unsatisfactory test results remind me that I am not studying.
3. When I encounter difficulties in learning, it may mean that I am not good at learning, and it would be better to switch to other things.
4. Sometimes, it feels too difficult to learn—it is even impossible to learn well. This may be a good thing, because it makes me understand that I should do other things.
5. Students should not waste time on things that do not make sense to them. If they feel that something is too difficult, they should try to do things that they can do successfully. *
6. As a student, if I think learning is too difficult, it may mean that I am not good at learning.

**Proportion of Missing Data**

To maximize our sample, we imputed missing data using the R mice package (Multivariate Imputation by Chained Equations; van Buuren & Groothuis-Oudshoorn, 2011). Following recommendations by Enders (2017) and Mainzer and colleagues (2021), we imputed missing values at the item-level and created unique multiple imputation models for each missing variable. We used a mixed item-scale approach for determining predictors of each multiple imputation model (for details, Mainzer et al., 2021). Data imputation focused on the analytic dataset. We did not impute descriptive variables. For data transparency purposes, we present the proportion of missing data prior to imputation for each analytic variable in Table S4. Following Madley-Dowd and colleagues (2019), large proportions of missing data are not problematic when multiple imputation is implemented correctly.

**Table S4**

*Percentage of Missing Data for Each Analytic Variable at Each Time Point*

| Scale | T0 | T1 | T1+ | T2 | T2+ | T3 |
| --- | --- | --- | --- | --- | --- | --- |
| Difficulty-as-Importance | -- | 9.05 | -- | 11.86 | -- | 42.05 |
| Difficulty-as-Impossibility | -- | 7.34 | -- | 10.76 | -- | 41.57 |
| Accepting Fate | -- | 9.90 | -- | 12.10 | -- | 42.30 |
| Optimism for the Future | -- | 7.58 | -- | 10.64 | -- | 40.83 |
| Academic Possible Identity Certainty | -- | 5.63 | -- | 8.19 | -- | 39.85 |
| Prior Academic Scores | 8.44 | -- | -- | -- | -- | -- |
| High-Stakes Test Scores | -- | -- | 8.44 | -- | -- | -- |
| School Placement | -- | -- | -- | -- | 40.95 | -- |

*Note.* All numbers are percentages.

**Preliminary Measure Analyses**

In Tables S5 to S7, we provide detailed results from our preliminary measure analyses. We summarize the common “rules of thumb” for model fit indices to facilitate the interpretation of results (Hu & Bentler, 1999; Schermelleh-Engel et al., 2003).

- **Chi-squared statistic** **(χ^2^)**: lower values indicate better model-data fit (i.e., the hypothesized model deviates less from a perfect, saturated model). The test is sensitive to sample size, such that large sample sizes will often result in a statistically significant difference, even if that difference is small.
- **Root Mean Square Error of Approximation (RMSEA):** lower values indicate better fit, where values below 0.08 are typically regarded as acceptable and values below 0.05 are considered good fit.
- **Standardized Root Mean Square Residual (SRMR)**: lower values indicate better fit, where values below 0.10 are typically regarded as acceptable and values below 0.05 are typically regarded as good fit.
- **Comparative Fit Index (CFI) and Tucker-Lewis Index (TLI)**: higher values indicate better fit, where values above 0.90 are typically regarded as acceptable and values above 0.95 are typically regarded as good fit. The CFI and TLI are nearly identical, though the CFI is less sensitive to sample size than the TLI.
- **Akaike Information Criterion (AIC) and Bayesian Information Criterion (BIC)**: lower values indicate better fit, but these values should only be used for comparing hypothesized models. Hence, there is no “rule of thumb” cutoff value. The AIC and BIC are nearly identical, though the BIC penalizes model complexity more than the AIC.

***Confirmatory Factor Analysis: Difficulty Mindsets***

We estimated four confirmatory factor analysis models using T1 data to confirm that each difficulty mindset should be modeled as a separate construct. First, we compared three models: a one-factor model in which all items were forced to load onto one construct, a two-factor model in which the task-based difficulty mindsets were forced to load onto one construct while the life-based difficulty mindsets were forced to load onto another construct, and a four-factor model in which each mindset was forced to load onto its own construct. While the four-factor model was the better fitting model of the three, model fit was not acceptable. To remedy the poor fit, we dropped scale items with loadings less than .6 and fit a fourth, revised four-factor model. Specifically, we dropped one of six items from the difficulty-as-impossibility scale, three of ten from the acceptance of fate scale, and three of seven from the optimism scale. As can be seen in Table S5, the revised four-factor model had acceptable fit, with CFI > .90, SRMR < .08, RMSEA < .08, and lower AIC, BIC, and χ^2^ values (Hu & Bentler, 1999; Schermelleh-Engel et al., 2003).

**Table S5**

*Difficulty Mindset Scales: Confirmatory Factor Analysis Using T1 Scores*

| Model | CFI | SRMR | RMSEA [90% CI] | AIC | BIC | χ^2^ | *df* |
| --- | --- | --- | --- | --- | --- | --- | --- |
| 1-Factor Model | .58 | .10 | .11 [.11, .11] | 60670 | 60942 | 3278.13 | 377 |
| 2-Factor Model | .71 | .09 | .09 [.09, .09] | 59484 | 59762 | 2394.53 | 376 |
| 4-Factor Model | .89 | .06 | .06 [.05, .06] | 57900 | 58201 | 1117.57 | 371 |
| Revised 4-Factor Model | .94 | .04 | .05 [.05, .05] | 44089 | 44334 | 606.04 | 224 |

*Note.* CI = confidence interval. Models were estimated with maximum likelihood estimation with robust (Huber-White) standard errors and missing values were handled with multiple imputation before model estimation.

***Confirmatory Factor Analysis: Identity Certainty and Strategy Certainty***

We estimated two confirmatory factor analysis models using T1 data to confirm that certainty of attaining academic possible identities and certainty of using effective strategies to attain academic possible identities should be modeled as separate constructs. We compared two models: a one-factor model in which all items were forced to load onto one construct and a two-factor model in each measure was forced to load onto its own construct. As can be seen in Table S6, the two-factor model was the better model with greater CFI and lower SRMR, RMSEA, AIC, BIC, and χ^2^ values (Hu & Bentler, 1999; Schermelleh-Engel et al., 2003).

**Table S6**

*Identity Certainty and Strategy Certainty: Confirmatory Factor Analysis Using T1 Scores*

| Model | CFI | SRMR | RMSEA [90% CI] | AIC | BIC | χ^2^ | *df* |
| --- | --- | --- | --- | --- | --- | --- | --- |
| 1-Factor Model | .90 | .06 | .16 [.14, .18] | 20479 | 20545 | 172.46 | 14 |
| 2-Factor Model | .97 | .03 | .08 [.06, .11] | 20267 | 20337 | 57.60 | 13 |

*Note.* CI = confidence interval. Models were estimated with maximum likelihood estimation with robust (Huber-White) standard errors and missing values were handled with multiple imputation before model estimation.

***Longitudinal Measurement Invariance***

We tested configural variance (no constraints), metric invariance (factor loadings constrained to be equal across time), and scalar invariance (factor loadings and item intercepts constrained to be equal across time). As can be seen in Table S7, each configural model had at least acceptable fit, with CFI > .90, SRMR < .08, RMSEA < .08 (Hu & Bentler, 1999; Schermelleh-Engel et al., 2003). As interpretations of χ^2^ difference tests are subject to the same flaws as interpretations of fit based on χ^2^ tests (Chen, 2007; Cheung & Rensvold, 2002), we followed Chen’s (2007) recommendation to evaluate changes in CFI supplemented by changes in SRMR or RMSEA. Specifically, we used the following cutoff criteria for measurement invariance: changes in fit criteria between the configural model and the metric model indicate non-invariance when there is a change of ≥ -.010 in CFI supplemented by a change of ≥ .015 in RMSEA or a change of ≥ .030 in SRMR; changes in fit criteria between the metric model and the scalar model indicate non-invariance when there is a change of ≥ -.010 in CFI supplemented by a change of ≥ .015 in RMSEA or a change of ≥ .010 in SRMR. As can be seen in Table S7, we found metric and scalar invariance for all difficulty mindset measures, identity certainty, and strategy certainty.

**Table S7**

*Testing Longitudinal Measurement Invariance Over Time for Repeated Measures*

| Mindset | Model | *df* | χ^2^ | *p* | CFI (Δ) | RMSEA (Δ) | SRMR (Δ) | AIC | BIC |
| --- | --- | --- | --- | --- | --- | --- | --- | --- | --- |
| Difficulty-as-Importance | | |  |  | |  |  |  |  |
|  | Configural | 120 | 218.50 | <.001 | 0.98 | 0.03 | 0.03 | 29763 | 30088 |
|  | Metric | 130 | 229.77 | <.001 | 0.98 (.00) | 0.03 (.00) | 0.03 (.00) | 29753 | 30031 |
|  | Scalar | 140 | 274.90 | <.001 | 0.97 (-.01) | 0.03 (.00) | 0.03 (.00) | 29787 | 30018 |
| Difficulty-as-Impossibility | | |  |  |  |  |  |  |  |
|  | Configural | 77 | 320.68 | <.001 | 0.95 | 0.06 | 0.04 | 25054 | 25327 |
|  | Metric | 85 | 358.06 | <.001 | 0.94 (-.01) | 0.06 (.00) | 0.06 (.02) | 25086 | 25321 |
|  | Scalar | 93 | 391.23 | <.001 | 0.93 (-.01) | 0.06 (.00) | 0.06 (.00) | 25101 | 25298 |
| Accepting Fate | |  |  |  |  |  |  |  |  |
|  | Configural | 233 | 867.00 | <.001 | 0.93 | 0.06 | 0.04 | 43433 | 43861 |
|  | Metric | 247 | 901.78 | <.001 | 0.93 (.00) | 0.06 (.00) | 0.04 (.00) | 43445 | 43807 |
|  | Scalar | 261 | 995.18 | <.001 | 0.92 (-.01) | 0.06 (.00) | 0.04 (.00) | 43522 | 43818 |
| Optimism for the Future | |  |  |  |  |  |  |  |  |
|  | Configural | 43 | 131.93 | <.001 | 0.97 | 0.05 | 0.03 | 2499 | 25217 |
|  | Metric | 49 | 149.11 | <.001 | 0.97 (.00) | 0.05 (.00) | 0.04 (.01) | 25002 | 25195 |
|  | Scalar | 55 | 184.07 | <.001 | 0.96 (-.01) | 0.05 (.00) | 0.04 (.00) | 25028 | 25192 |
| Academic Possible Identity Certainty | | | |  |  |  |  |  |  |
|  | Configural | 18 | 76.94 | <.001 | 0.98 | 0.06 | 0.03 | 24043 | 24212 |
|  | Metric | 22 | 84.38 | <.001 | 0.98 (.00) | 0.06 (.00) | 0.03 (.00) | 24041 | 24192 |
|  | Scalar | 26 | 104.08 | <.001 | 0.98 (.00) | 0.06 (.00) | 0.03 (.00) | 24053 | 24185 |
| Using Academic Strategies Certainty | | | | |  |  |  |  |  |
|  | Configural | 43 | 163.76 | <.001 | 0.97 | 0.06 | 0.03 | 33586 | 33807 |
|  | Metric | 49 | 180.49 | <.001 | 0.96 (-.01) | 0.06 (.00) | 0.03 (.00) | 33589 | 33782 |
|  | Scalar | 55 | 195.10 | <.001 | 0.96 (.00) | 0.06 (.00) | 0.03 (.00) | 33587 | 33752 |

*Note.* CFI and TLI are nearly identical, so we report only CFI. Δ = “change in”. Models were estimated with maximum likelihood estimation with robust (Huber-White) standard errors and missing values were handled with multiple imputation before model estimation.

**References**

Chen, F. F. (2007). Sensitivity of goodness of fit indexes to lack of measurement invariance. *Structural Equation Modeling, 14,* 464-504. https://doi.org/10.1080/10705510701301834

Cheung, G. W., & Rensvold, R. B. (2002). Evaluating goodness-of-fit indexes for testing measurement invariance. *Structural Equation Modeling, 9,* 233-255. https://doi.org/10.1207/S15328007SEM0902_5

Enders, C. K. (2017). Multiple imputation as a flexible tool for missing data handling in clinical research. *Behaviour Research and Therapy*, *98*. https://doi.org/10.1016/j.brat.2016.11.008

Hu, L., & Bentler, P, M. (1999). Cutoff criteria for fit indexes in covariance structure analysis: Conventional criteria versus new alternatives. *Structural Equation Modeling, 6,* 1-55. https://doi.org/10.1080/10705519909540118

Madley-Dowd, P., Hughes, R., Tilling, K., & Heron, J. (2019). The proportion of missing data should not be used to guide decisions on multiple imputation. *Journal of Clinical Epidemiology, 110,* 63-73. https://doi.org/10.1016/j.jclinepi.2019.02.016

Mainzer, R., Apajee, J., Nguyen, C. D., Carlin, J. B., & Lee, K. J. (2021). A comparison of multiple imputation strategies for handling missing data in multi‐item scales: Guidance for longitudinal studies. *Statistics in Medicine, 40*. https://doi.org/10.1002/sim.9088

Rigdon, E. (1996). CFI versus RMSEA: A comparison of two fit indexes for structural equation modeling. *Structural Equation Modeling, 3,* 369-379. https://doi.org/10.1080/10705519609540052

Schermelleh-Engel, K., Moosbrugger, H., & Müller, H. (2003). Evaluating the fit of structural equation models: Tests of significance and descriptive goodness-of-fit measures. *Methods of Psychological Research, 8,* 23-74.

van Buuren, S., & Groothuis-Oudshoorn, K. (2011). “mice: Multivariate Imputation by Chained Equations in R.” *Journal of Statistical Software*, *45*. https://doi.org/10.18637/jss.v045.i03
