# Supplementary material for: Believing that difficulty signals importance improves school outcomes by bolstering academic possible identities, a recursive analysis
Source: PLoS One. 2024 Oct 21;19(10):e0308376. doi: 10.1371/journal.pone.0308376 (PMC11493252; doi:10.1371/journal.pone.0308376)
Supplement: Attachment — Submitted filename: Response to Reviewers.docx [file pone.0308376.s003.docx]

Author responses are highlighted in yellow.

**Academic Editor:**

Journal Requirements:

When submitting your revision, we need you to address these additional requirements.

1. Please ensure that your manuscript meets PLOS ONE's style requirements, including those for file naming. The PLOS ONE style templates can be found at

[https://journals.plos.org/plosone/s/file?id=wjVg/PLOSOne_formatting_sample_main_body.pdf](https://urldefense.com/v3/__https://journals.plos.org/plosone/s/file?id=wjVg*PLOSOne_formatting_sample_main_body.pdf__;Lw!!LIr3w8kk_Xxm!v8SCqdDyueyPiCImM5jJgTnd-BZ9N_LzgzWMoWopcrEMDJqXd_6-Y6Zfoyi6WqQpnClLfqmfZ6F9ge6D$) and

[https://journals.plos.org/plosone/s/file?id=ba62/PLOSOne_formatting_sample_title_authors_affiliations.pdf](https://urldefense.com/v3/__https://journals.plos.org/plosone/s/file?id=ba62*PLOSOne_formatting_sample_title_authors_affiliations.pdf__;Lw!!LIr3w8kk_Xxm!v8SCqdDyueyPiCImM5jJgTnd-BZ9N_LzgzWMoWopcrEMDJqXd_6-Y6Zfoyi6WqQpnClLfqmfZ9-lUV-G$)

Thank you. We have updated the formatting in our manuscript to adhere to the PLOS ONE style requirements. We have also edited the supplemental materials file name to be “S1_File.docx” and note it in the “Supporting information” section of the manuscript.

2. Please include a complete copy of PLOS’ questionnaire on inclusivity in global research in your revised manuscript. Our policy for research in this area aims to improve transparency in the reporting of research performed outside of researchers’ own country or community. The policy applies to researchers who have travelled to a different country to conduct research, research with Indigenous populations or their lands, and research on cultural artefacts. The questionnaire can also be requested at the journal’s discretion for any other submissions, even if these conditions are not met. Please find more information on the policy and a link to download a blank copy of the questionnaire here: <https://journals.plos.org/plosone/s/best-practices-in-research-reporting>. Please upload a completed version of your questionnaire as Supporting Information when you resubmit your manuscript.
We completed the questionnaire on inclusivity in global research and uploaded it as “S2_File.docx.” As requested in the questionnaire’s directions, we also now include an “Inclusivity in global research” subsection in the “Methods” section.

3. Please include your full ethics statement in the ‘Methods’ section of your manuscript file. In your statement, please include the full name of the IRB or ethics committee who approved or waived your study, as well as whether or not you obtained informed written or verbal consent. If consent was waived for your study, please include this information in your statement as well.
Thank you. We now include an ethics statement in the “Methods” section.

4. Please review your reference list to ensure that it is complete and correct. If you have cited papers that have been retracted, please include the rationale for doing so in the manuscript text, or remove these references and replace them with relevant current references. Any changes to the reference list should be mentioned in the rebuttal letter that accompanies your revised manuscript. If you need to cite a retracted article, indicate the article’s retracted status in the References list and also include a citation and full reference for the retraction notice
To the best of our knowledge, nothing we cited was later retracted. We note that to address reviewer comments, especially regarding use of our measures in Chinese samples, we expanded our references.

**Reviewer #1:**
Review Comments

This study examined improves school outcomes by bolstering school-focused possible identities through multi-stage data collection and analysis, and the research topic chosen is of some significance, but there are some issues that need to be considered by the authors. The details are as follows:

I. Abstract

1. The keywords do not highlight the variables that are the focus of the article; please make appropriate deletions and highlight key variables.
Thank you. We have revised the keywords with a focus on using the names of our key variables.

II. Introduction

1. The author presents "A recent review of the voluminous literature on possible future selves and identities [3] documents a variety of conditions under which an accessible future self matters for behaviour". which an accessible future self matters for behaviour", from the whole introduction, there is no review of the previous literature, reflect and find contradictions, and then lead to the research purpose of this paper, that is to say, the part of the introduction is a little bit thin, logic and relevance is not strong.
Thank you. We revised the “Identity-based motivation theory” section so that the review of the literature is properly sub-headed and we provide more detail on this review. The review just came out (2023) and does indeed include all references to future self or possible self or possible identity and some measure of behavior. Because it is so broad, it does highlight gaps that would otherwise not be noticed. Here is the description of the methods section from the paper:

“We chose January 1985 as our starting point, just before Markus and Nurius’s (1986) seminal paper on possible selves. We searched the PsychINFO database abstracts with the terms: “future self/ves,” “possible identity/ies,” “possible self/ves,” and “mental contrasting^a^” and added ancestry searches and searches based on known authors. We pulled papers from our starting point through January 27, 2022. We read abstracts and then the full studies of all papers with abstracts suggesting that the authors measured or systematically shifted focus on future me or a specific possible identity and measured a future-focused behavior or intention to act. We included studies that assessed changes in physiological measures connected to health—cortisol and blood pressure but excluded studies focused solely on mapping to regions of the brain (e.g., Tanguay, Palombo, Atance, Renoult, & Davidson, 2020). Table 2 shows our yield of 101 papers describing 170 studies and 205 results, sorted by approach (possible self, self-gap, and self-continuity). As Table 2 reveals, 4 in 10 studies focused on possible selves (37.6%) and self-continuity (40.6%).”

^a^We focused on behavior rather than outcomes such as affect, optimism, or depression, for which other more targeted reviews are available (e.g., Carrillo et al., 2019; Heekerens & Eid, 2021, meta-analyses of best possible self writing studies using non-behavioral dependent variables; Schubert, Eloo, Scharfen, & Morina, 2020 meta-analysis of the effect of positive and negative future selves on positive and negative affect). We included mental contrast studies that use a behavioral dependent variable if the future and present seem self-like, though not specified as being about the self. We excluded studies that combined a mental contrast with something else if the mental contrast effect could not be separated out. For example, a large number of studies combine mental contrast with writing implementation intentions, writing a set of contingencies linking cues (if) to behaviors (then), effects may be due to having implementation intentions or to mental contrast or to some combination of both (see, Cross & Sheffield, 2019 for a meta-analysis of mental contrasting with and without adding implementation intentions for health behaviors).

2. Following up on the previous comment, can one piece of literature summarise all previous research findings? Please ask the authors to consolidate and organise the latest literature.
We appreciate the comment. The review did indeed have the intention of summarizing the full literature, as noted above in the methods section we pulled from the paper. Given that the review is both current and thorough, summarizing that provides a comprehensive survey of the literature.

3. At the end of the introductory section, the authors are requested to briefly state the value and significance of the study while briefly outlining the purpose of the study in this paper.
We appreciate the comment and revised our manuscript such that the section “Gaps to be addressed” more clearly states the research gaps our study addresses.

III. Cultural context and theoretical elaboration section

1. Why do Chinese students feel academic pressure? Lack of arguments presented.
We appreciate the comment and revised our manuscript so that our “High-stakes school transitions in China and around the world” section better represents our argument and line of reasoning.

2. Based on Chinese culture, two possible student perceptions were explained: optimism and resignation. However, this kind of mentality is not only found in the Chinese student population, but is also a phenomenon that exists in all countries, and if Chinese students were selected as subjects for the study based on this statement, the rationality seems to need to be rethought. In addition, this passage still does not explain optimism and resignation enough, but only mentions them briefly through the literature, which is not deep enough, and does not really elaborate on the differences in students' academic performance due to their different perceptions of optimism and resignation to account for them.
We appreciate the comment and address it in two ways. First, we drop the culture-based label and clarify that these beliefs are culturally-available for Chinese *and* other students and that the literature documents it among children in China from the age of 9 years. The literature on possible or future selves includes both Western and Chinese samples so our research contributes to this body of work. Second, we expanded the newly named “Optimism and accepting fate” section to operationalize and situate each construct.

3. The content of this part of "Identity-based motivation theory" can be properly integrated with the introduction, and part of it can be put into the introduction. At present, the content of this part of the expression gives the feeling that it is too long, please make appropriate reduction, highlighting the logic of the language, rather than a large number of words and the accumulation of literature.
We appreciate this comment and respond by providing revised headings, taking out redundancies, and adding subheadings so that the unique content of each subheading stands out.

IV. Methodological component

1. The author chose the primary to junior high school level, why did he choose this level of students? Reading your article, it is partly explained that only half of the students can go on to higher education, which means that there is a high degree of difficulty in this kind of higher education. However, the reality is that in the current Chinese context, only half of all promotions occur in the third year of junior high school and the first year of high school.
Thank you for this question. We have revised the “High-stakes school transitions in China and around the world” section to better represent our argument and line of reasoning. Regarding the point that “only half of all promotions occur in the third year of junior high school and the first year of high school” we are not sure we understand this comment’s meaning–are you saying that even students who make it through to a good secondary school may not go further?

2. there are large regional variations in education in China, and the regional locations of the sample schools taken by the researcher should be described.
Thank you for noting this omission. We added a section titled “Participants” and included the province, Guangdong Province. We do not name the city as the schools wished to remain anonymous and the member of our author team who collected this data assured them that the city name would not be used.

3. please put the data of demographic variables into the research methodology section through textual representation.
Thank you for this comment. We have reframed how we present the demographics of our sample in the newly named and organized “Participants” section.

4. The author collected a total of 818 subjects, so how many questionnaires were distributed at each stage? What was the recovery rate? How many were lost at each stage? What data were removed? What was the basis? None of this is seen in the article.
Thank you for noting this inadvertent omission. Assenting students for whom we had parental consent completed the questionnaires in class. Data loss across time points was due to the fact that not all students present at T1 were in the classroom at T2 and/or T3, and some were present at T2 but not T3, and the reverse.We are not using the term “recovered” because the research team members who collected questionnaires remained in the classroom while students filled them out–questionnaires were not “recovered” in the sense that we did not hand out blank questionnaires and ask for them to be dropped off or returned at a later time. We now share the number of questionnaires obtained at each time point in the “Participants” section. The percentage of questionnaires obtained at each time point was 94%, 92%, and 60%, respectively.

5. Sampling: the authors mention that there were 15 schools and then 5 recruitment letters were distributed and 3 schools agreed to collect data? How did the authors manage to select 5 schools out of the 15 and distribute 5 recruitment letters? This does not seem to be explained in detail in the article.
We include this detail in the first paragraph of the newly named and organized “Procedure” section: “To obtain an adequate sample size, we recruited three schools by sending recruitment letters one by one to randomly selected schools, sending five letters in total.”

6. The authors have translated the English scale, what is the reliability and validity of the translated scale? Does it meet the requirements? The authors did not provide details on whether the scales could be used to measure students in the Chinese cultural context.
We thank you for this comment. In the Measures section (Table 2), we now share the Cronbach's alpha reliability coefficients for each of our measures (values ranged from .79 to .93) and note the initial Chinese use of scales that have already been used in China. We also added a Construct Validity section to our Results.

7. The authors did not report the reliability coefficients for each scale.
We thank you for alerting us, we now share this in the Measures section, Table 2.

8. Please make appropriate deletion and integration of the three parts of "Analysis plan, Preliminary analysis, Main analysis", which can be clearly stated.
Thank you for this comment. We have now deleted the subheadings under “Analysis plan” and integrated the contents into one section.

9. Please report in detail the structural and discriminant validity ( χ2/df, CFI, TLI, RMSEA, SRMR) etc. between variables.
Thank you for this comment. We detail the above requested measures of fit in Supplemental Materials Tables S4 and S5 and summarize the results of these analyses in more detail in the newly reorganized Construct Validity subsection of Preliminary Results.

10. For the sample description section, please make deletions; this section is also written in a way that is not consistent with the writing style of the research methods section.
Thank you for this comment. We revised to move this descriptive information to the Participants subheading in Supplemental Materials. We collected this descriptive information to provide a snapshot of the children in our study particularly for readers not familiar with China.

11. Please make deletions from the description of the sample, which is also not in line with the writing style of the research methodology section.
Thank you, see above, where we believe we address the issue raised here.

V. Discussion section

1. the results of the study are not expanded upon, just repeating the results of the previous study, it is the in-depth discussion and digging into the results of the study that is the most valuable part.
We appreciate your comment. As revised, in the first paragraph of our Discussion, we summarize our results as is common in the social and developmental psychology literature as a way of orienting the reader to what we found. Then we connect our results to the broader literature and highlight the implications of our results.

2. the discussion section is rather thin, as if it were a conclusion, not a discussion.
We appreciate your comment. As revised, we provide a more detailed connection between our results and the broader literature and highlight the implications of our results.

VI. Conclusion

1.The discussion is mixed and has a conclusion, which can be abridged, and the focus is on enriching the discussion section.
We appreciate your comment. As revised, the conclusion section has two sentences to focus the reader on our sense of the main contribution or punchline of our results. The discussion highlights implications in three literatures. We added more descriptive implications in each of these sections.

**Reviewer #2:**
I have now had a chance to read MS PLOS-D-24-06508. Foremost, I saw a great deal to like about this article. In particular, I found the focus on reciprocal pathways to be a refreshing and exciting departure from most work that focuses on unidirectional effects. I also like that this work takes a novel yet programmatic approach to understanding the dynamic links among possible self-relevant variables in an important naturalistic context. Having said that, I did have a few minor suggestions/questions to guide the authors’ revision.

1. The authors need to define key terms (possible identities, certainty) up front. As an example of where this might matter, the authors discuss both optimism and certainty. Even though I agree that there is a meaningful difference between these two constructs, this distinction may elude naïve readers without clear definitions of both. In fact, it was only in the methods section that I was able to discern that the authors were referring to dispositional and not situated optimism (see Armor and Taylor, 1998). Thus, I would recommend clearly defining variables up front, particularly every variable represented in your path model.
Thank you for these helpful suggestions. We now include operationalizations of possible identity certainty, optimism, and fatalism in our revised introduction section and clarify that prior studies describe dispositional optimism, fatalism, and certainty.

2. Also, does the attainment of one’s ideal school placement represent the presumed “possible identity” here? If so, then, what exactly does the Time 3 certainty index represent given it is no longer about a “possible” but, instead, an already attained (or unattained) identity.
We realize our first submission was unclear. In the Measures section we now include the four items in the possible identity certainty scale (Supplemental Materials has these in Chinese). The ideal school placement question was asking students what secondary school they wanted to go to. We asked if they got into that school once they had been given this information. We asked this and other daily activity questions to get a descriptive snapshot of students in our sample. Following the suggestions of Reviewers 1 and 3, we moved this descriptive information that is not relevant to the core studied questions to Supplemental Materials.

3. Although the results are impressive, I was wondering what objective outcomes that things like T3 possible identity certainty might predict long-term.
Thank you for this comment. We showed that possible identity certainty predicts standardized test performance controlling for prior grades. Though we do not have a T4, we expect that high school performance will be incrementally affected by possible identity certainty. This is certainly a point for future research.

4. Why are Tables 6-7 included in the text and not with the rest of the tables at the end?
Thank you for this question. In the revision, we moved all Tables to the paper. Initially, we thought that the Appendix would be for descriptive information and the body of the paper for results. We realize that this logic was not clear.

5. Although they focused on links with the MC inference of “difficulty as importance”, I wondered what the predictions and findings would be for the MC inference of “difficulty as improvement”?
We appreciate the question. The difficulty-as-improvement literature we cite shows that people who endorse difficulty-as-improvement prefer effortful means of attaining their possible identities and disdain less effortful ones. Had the measure been available when data for this study were collected, we would also have included this measure.

**Reviewer #3:**
Overall, the strength of the paper is that it tried to examine the recursive process of two types of difficult mindsets (important vs. impossibility), school-focused possible identities, and school outcomes over a short longitudinal period (two-month study) with a relatively big sample size (N= 818). The downsides are the generalizability of the findings, given that the sample is Chinese students, and the presentation of research questions, results, and interpretations throughout the paper. The paper needs a major conceptual and structural revision in order to contribute to the current literature.

1. Since the research context is situated in China, it is hard to make a global claim worldwide; citing other countries' similar findings doesn't convince the generalizability of the study findings (Check the High-stakes school transitions in China and around the world section).
We appreciate your comment and concern. We dropped the “culture-based” terminology, revised our introduction to highlight why China is relevant but not necessarily unique (see also Reviewer 1, point #2), and added consideration of what might be new about studying Chinese students to our discussion section. Given that social scientists are trying to study constructs across a variety of societies, studying a phenomenon in the world’s second most populous country seems a good reason to include China.

2. Missing in-text citations (e.g., p. 7: The first literature focuses on self-continuity, the effects of including the future self in the current self (citation)).
Thank you for catching that confusing sentence. We revised so that it is clear that we are summarizing the results of the cited review.

3. Avoid causal language: boost, bolster, improve, etc. SEM tests the relationships among variables, and many alternative models can give similar/better model fits.
Thank you for this suggestion. We revised our title to add recursive analyses as the method we are using. We are controlling for the prior measurements in our analyses so that we are looking at a marginal ‘boost’ that is not just an association. We do not use casual language or the word boost in the results section. We do use this word in the conclusion section as it is the everyday language equivalent of saying that we are showing marginal increases controlling for the past. We are not sure how we would otherwise use language that clarifies that that is the finding (and not just a point in time association) and do not intend to say cause, just boost. Regarding the SEM approach, in the Discussion section we clarify that an SEM approach does not allow for causal inference. Similarly, the title provides the everyday language explanation of what we found: controlling for T1 Certainty in Attaining School-focused Possible Identities, T2 Certainty in Attaining School-focused Possible Identities is *higher* if T1 Difficulty-as-Importance is higher. The same is true at T3, controlling for T2 Certainty in Attaining School-focused Possible Identities, T3 Certainty in Attaining School-focused Possible Identities is *higher* if T2 Difficulty-as-Importance is higher. It is not causal it is however directional (higher) hence the word improve and the word bolster.

4. Instead of listing a lot of findings, summarize the most critical findings in the Abstract.
Thank you for this comment. We initially tried to include all of our procedures and findings in the abstract. We have revised the abstract to highlight in everyday language what the theory predicts, what is the gap in prior studies, what our method allows us to test, and what we found.

5. Research questions and hypotheses are missing in the Current Study section
Thank you for this comment. We can see that our initial submission lacked clarity as our research questions and hypotheses were in the “Summary and proposed model” section. We have now reformatted our hypotheses to improve readability and moved them to the “The current study” section.

6. What are the main focus(es) in the Research Questions and proposed models? You may reorganize the findings based on the main focus(es), such as recursive/unidirectional and direct/indirect relationships.
Thank you for this comment. Now that we have reformatted our hypotheses to improve readability and moved them to the current study section, we believe that the main focus is now easier to discern and easier to see how the Results address each of these.

7. The model fits for RMSEA (.08) and SRMR (.08) for the second model are not very good. I'd like to know about the modification index results. Also, report 90% CI for RMSEA (a common practice).
Thank you for your comment regarding reporting the 90% CI for RMSEA, we now do so for our Step 1 Model, our Step 2 Model, and our CFA analyses (detailed in the supplemental materials, Tables S6 and S7). Regarding the modification index results, we share below a table of the modification indices sorted by largest modification index value. For brevity, we share the first 20 rows only. We note that the lower RMSEA and SRMR is not surprising given the exploratory nature of our analyses, especially regarding optimism for the future and accepting fate, neither of which displayed consistent relationships with the key variables of interest. We collected these data in order to address the question of whether the theorized recursive paths described in IBM theory generalize to other constructs associated with academic achievement in the literature. While model fit would likely improve if we were to drop paths involving these variables from our analysis, given that we collected these data for a broader explanation, we retain them.

| lhs | op | rhs | mi | epc | sepc.lv | sepc.all | sepc.nox |
| --- | --- | --- | --- | --- | --- | --- | --- |
| t3_imposs_avg | ~ | t1_fatalism_avg | 64.448 | 0.220 | 0.220 | 0.227 | 0.227 |
| t1_sfpi_avg | ~ | t2_imposs_avg | 58.736 | 2.019 | 2.019 | 0.918 | 0.918 |
| t1_fatalism_avg | ~ | t3_imposs_avg | 57.352 | 0.254 | 0.254 | 0.247 | 0.247 |
| t2_import_avg | ~ | t3_imposs_avg | 47.825 | -0.214 | -0.214 | -0.224 | -0.224 |
| t1_fatalism_avg | ~~ | t3_imposs_avg | 45.510 | 0.070 | 0.070 | 0.188 | 0.188 |
| t1_imposs_avg | ~~ | t3_imposs_avg | 44.979 | -0.117 | -0.117 | -0.324 | -0.324 |
| t1_imposs_avg | ~~ | t2_imposs_avg | 39.620 | -0.129 | -0.129 | -0.331 | -0.331 |
| t3_imposs_avg | ~ | t3_fatalism_avg | 39.549 | 0.239 | 0.239 | 0.265 | 0.265 |
| t1_sfpi_avg | ~ | t2_import_avg | 37.871 | -2.070 | -2.070 | -0.882 | -0.882 |
| t1_fatalism_avg | ~~ | t2_fatalism_avg | 35.399 | -0.155 | -0.155 | -0.340 | -0.340 |
| t1_fatalism_avg | ~ | t2_sfpi_avg | 34.795 | -0.718 | -0.718 | -1.694 | -1.694 |
| t1_sfpi_avg | ~ | t2_fatalism_avg | 34.041 | 1.228 | 1.228 | 0.629 | 0.629 |
| t1_import_avg | ~~ | t2_import_avg | 30.660 | -0.166 | -0.166 | -0.478 | -0.478 |
| t3_imposs_avg | ~ | t3_optimism_avg | 29.918 | -0.197 | -0.197 | -0.239 | -0.239 |
| t2_import_avg | ~ | t2_imposs_avg | 29.525 | -0.316 | -0.316 | -0.337 | -0.337 |
| t2_import_avg | ~ | t1_imposs_avg | 29.525 | -0.170 | -0.170 | -0.180 | -0.180 |
| t3_imposs_avg | ~ | t2_fatalism_avg | 28.833 | 0.130 | 0.130 | 0.149 | 0.149 |
| t2_fatalism_avg | ~ | t2_optimism_avg | 27.507 | -0.241 | -0.241 | -0.264 | -0.264 |
| t2_fatalism_avg | ~ | t1_optimism_avg | 27.506 | -0.153 | -0.153 | -0.158 | -0.158 |
| t1_optimism_avg | ~ | t2_fatalism_avg | 27.312 | -0.196 | -0.196 | -0.190 | -0.190 |

8. The results section is difficult to understand because it lists a lot of information. More guiding summaries are necessary.

Thank you for this suggestion. We agree. We revised the content and presentation, as you can see, we now have appropriate organizing subheadings and moved what are actually descriptions of the sample, not of results, to Supplemental Materials.

9. Some sections are redundant (e.g., Sample and Method and Sampling).
Thank you for this comment. We have restructured the subsections within the “Method and Sampling” section to limit redundancy.

10. Some sections are not very relevant to the current study (e.g., Sample Description and Table 3).
Thank you for this comment. We agree, we moved this descriptive section to supplemental materials, as noted in the “Participants” section.

11. The figures need to include titles and be clear. I’d recommend deleting the non-significant paths and adding significant path results (i.e., standardized coefficients).
Thank you for your comments. We follow PLOS One guidelines and have the titles in the body, not in the Figure files –“Do not include… figure number/title/caption within figure files.” (<https://journals.plos.org/plosone/s/figures#loc-how-to-submit-figures-and-captions>). In Fig 3, we include only significant paths and control paths. Given the complexity of Fig 3, we could not clearly include each coefficient in the figure and instead report the exact path coefficients in the main body of the Results section and in Table 6 (previously numbered Table 7).

12. Table 7: I’d recommend omitting the non-significant results. Placing the predictor in the first column and the intermediate and the outcome variables in the next columns would help understand the relationship results.
We appreciate your suggestions. Please note that due to other revisions, Table 7 is now Table 6. Regarding the omission of non-significant results from Table 6, retaining non-significant results allows readers to understand the exact variables included in each path of the SEM. If we were to omit the non-significant results, readers may incorrectly infer that the omitted variables were not part of the analysis. Regarding the column structure of Table 6, we renamed the “Effect” column “Predictor Variable.” We think “intermediate” variables refer to difficulty-as-importance, difficulty-as-impossibility, optimism for the future, and accepting fate. We do not separate these variables into a separate “Intermediate Variable” column; given the nature of our SEM path analysis, these variables can be predictors or an outcome.

13. General writing style: Revise complex sentences that include too many meanings/explanations (e.g., Abstract: Identity-based motivation theory predicts that how well students do in school and how certain they are of attaining. . . . when school tasks and goals feel hard to think about or do.).
Thank you for this suggestion. We have revised complex sentences to increase ease and readability.
